# HIV drug resistance profile of HIV-1 CRF 01_AE protease and integrase coding regions in HIV infected Cambodian patients failing LPV-based 2^nd^ line antiretroviral regimen

**DOI:** 10.1186/1753-6561-5-S1-P89

**Published:** 2011-01-10

**Authors:** Janin Nouhin, Sopheak Ngin, Sreymom Ken, Vara Ouk, Olivier Segeral, Kimlay Chea, Kerya Phon, Jean-François Delfraissy, Eric Nerrienet

**Affiliations:** 1 HIV/Hepatitis Laboratory, Institut Pasteur du Cambodge, Phnom Penh, Cambodia; 2 Esther Program, Calmette Hospital, Phnom Penh, Cambodia; 3 Clinical Immunology Department, Bicêtre Hospital, Kremlin Bicêtre, France; 4 ANRS, Paris, France

## Background

By the end of 2009, the number of HIV-1 infected patients on RTI-based 1^st^ line and PI-based 2^nd^ line ARV regimen in Cambodia reached 34,000 and 1,500, respectively. We already reported good virological and immunological responses after 1 to 4 years in cohorts of patients on 1^st^ line and more recently, among an Esther cohort of 70 patients after 2 years on LPVr-based 2^nd^ line regimen. However, emergence of LPV resistant associated mutations is becoming a major concern in low and middle income countries.

## Objective

This study aimed to describe the resistance pattern of both the protease (PR) and integrase (IN) coding regions in HIV-1 CRF01-AE infected patients failing LPV-based 2^nd^ line regimen in Cambodia.

## Methods

Analysis of the Protease and Integrase drug resistance genotyping of 95 HIV-1 strains infected patients presenting detectable viral load on LPV/r-based 2^nd^ line regimen in Cambodia.

## Results

Lack of amplification in PR gene was observed for 18/95 presenting low viral load (median VL: 2.9Log_10_ copies/ml [IQR: 2.8-3.4]). The 77 other CRF01_AE strains, harbored polymorphism mutation in position M36, H69 and L89 conferring possibly resistance to TPV/r. Forty-nine (median VL: 5Log_10_ copies/ml [IQR: 4 - 5.5]) did not present any other PI associated resistance mutation. In contrast, 28 patients showed multiple resistances to PI. The median duration on LPV/r regimen was 34.5 months [IQR: 23.5 – 53.3] and the median VL was 5Log_10_ copies/ml [IQR: 4.3-5.6]. Twenty-five patients were resistant to LPV/r (7 possibly resistant). Twenty-seven were resistant to IDV, 21 and 19 to ATV/r and FPV/r, respectively. Twenty-five were resistant to NFV (10 possibly), 22 resistant to SQV/r (9 possibly). Seven showed resistance to DRV/r (5 possibly). Finally, excluding possible resistance, 21/28 (75%) was resistant for at least 3 PIs. Clinical investigation revealed that most of these 28 patients starting several RTIs and PIs early around 2000. All of them were sensitive to raltegravir, elvitegravir (integrase inhibitors), and etravirine (Non-Nucleoside reverse transcriptase inhibitorse).

## Conclusion

This study indicates that 28/95 (29.5%) of Cambodian patients presenting detectable viral load on LPV/r–based 2^nd^ line regimen developed resistance mutation for a large number of PIs. Most of them were not naïve for PI before LPV/r initiation. These results highlight an urgent need to evaluate the efficacy of LPV/r-based 2^nd^ line regimen at the national levels, allowing to design of a next 3^rd^ line ARV regiment in low and middle income countries.

